# Nutritional Status and Psychological Impairment in Rural Adolescent Girls: Pilot Data From “KOKAN” Region of Western India

**DOI:** 10.3389/fpubh.2018.00160

**Published:** 2018-06-21

**Authors:** Suvarna Patil, Charudatta Joglekar, Maruti Desai, Arvind Yadav, Swati Sonawane, Rupali Chavan, Rachana Mohite

**Affiliations:** ^1^Department of Medicine, Regional Centre for Adolescent Health and Nutrition, BKL Walawalkar Rural Medical College Chiplun, India; ^2^Statistics Unit, Regional Centre for Adolescent Health and Nutrition, BKL Walawalkar Rural Medical College Chiplun, India; ^3^Department of Biochemistry, Regional Centre for Adolescent Health and Nutrition, BKL Walawalkar Rural Medical College Chiplun, India; ^4^Department of Psychiatry, Regional Centre for Adolescent Health and Nutrition, BKL Walawalkar Rural Medical College Chiplun, India; ^5^Department of Dietetics, Regional Centre for Adolescent Health and Nutrition, BKL Walawalkar Rural Medical College Chiplun, India

**Keywords:** adolescent, psychological health, micronutrients, macronutrients, Y-PSC

## Abstract

**Background:** Adolescence is a period during which psychological foundations are laid down as well as consolidated. Not much information is available on rural Indian adolescent girls and their psychological health.

**Methods:** We did a pilot survey of psychological health of 80 adolescent girls residing at KOKAN region of western India. Psychological health was evaluated using Youth Paediatric Symptom Checklist (Y-PSC) consisting of 35 items with maximum score of 70. Girls with a score >30 were classified as psychologically impaired. In addition we also collected random blood sample and measured the micronutrients. Macronutrient intake was estimated by 24 h recall.

**Results:** The mean age of the girls was 14 years with a standard deviation of 1.5. In all 35/76 (46.1%) could be classified as psychologically impaired. There was a high prevalence of micronutrient deficiencies with varying degrees. More than 65% were deficient in calcium, zinc and folic acid. About 22% were anemic and 36% were vitamin B_1_ deficient. More than 75% had a low recommended dietary allowance (RDA) of macronutrients. Those with poor serum calcium concentration had higher psychological score (*p* < 0.05). Fat and calcium intakes were inversely associated with psychological score (*p* < 0.05 and *p* < 0.001 respectively). Odds ratios for psychological impairment were significant for those with low calcium levels [1.47 (95% CI 1.21, 4.31)], and for those with low calcium intake 1.43 (1.08, 3.19) and low iron intake 3.04 (1.02, 9.26).

**Conclusion:** Our pilot data has shown the urgent need to improve the nutrition of adolescent girls, which could improve their psychological health.

## Introduction

Adolescence is the stage of life in which a child transitions into an adult. It is characterized by accelerated growth, sexual maturation, and an increase in the complexity of psycho-social interactions. During adolescence physical, psychological and social foundations are laid and consolidated. More than half of the world's adolescent population live in Asia. India has the largest population of adolescents^1^. In many developing countries more than 50% of the adolescents fail to achieve their growth potential due to inadequate nutrition and faulty dietary habits (1, 2). Seeds of many psychological disturbances in adulthood are sown in adolescence. Adolescent children face many obstacles for healthy living. Most of the healthcare services in India are designed either for adults or children. Adolescent girls form a crucial segment of the population and as prospective mothers they form a vital bridge between the present and next generation. In India the prevalence of malnutrition among the girls remains very high (3). Adolescent girls are particularly vulnerable to poor nutrition and anemia (4–8). They face multiple barriers of gender discrimination, superstitions and social norms. This in turn affects their learning, cognitive function, and scholastic performance (9).

BKL Walawalkar Hospital is located at Dervan village situated about 250 km south of Mumbai. The area is a part of coastal region of “KOKAN.” In the “KOKAN” region, malnutrition posed a variety of threats to women and young girls. Malnutrition weakened women's ability to survive childbirth, and made them more susceptible to infections with fewer reserves to recover from illness. In 2010 the hospital launched a scheme to empower young women in the surrounding areas with the aims of making them confident to face challenges of life and capable of becoming healthy mothers. The scheme comprised of 3 programs consisting of:

1. screening for hemoglobin in adolescent girls of 11–16 years of age from schools
2. 3–5 days of residential camps to provide holistic education
3. Physical and nutritional assessment.

In a sample of adolescent girls we measured the nutrients in their blood and evaluated their psychological health to test the hypothesis that poor nutritional status will be associated with poor psychological health.

## Methods

Our hospital covered 30 schools as a part of empowerment of young women program in 2016. We randomly selected a school and listed out all the 297 adolescent girls. Then we randomly selected 80 adolescent girls and enrolled them in our study. They were brought to the hospital as part of empowerment program. During their stay at the hospital, a random blood sample was collected and they underwent various investigations and assessments.

### Anthropometry

The height and weight of the adolescent girls was recorded and Body Mass Index (BMI) was calculated. International Obesity Task Force (IOTF) standards (10) were used to classify them into various categories of thinness and obesity. Stunting and severe stunting was defined using WHO reference curve for height^2^.

### Psychological evaluation

All the subjects in our study filled Youth Paediatric Symptom Checklist (Y-PSC) (11, 12). This test is a tool used t to recognize cognitive, emotional, and behavioral problems in adolescents. It consists of 35 items each of which is rated “Never,” “Sometimes,” or “Often” *t* and scored 0, 1, and 2 respectively. The test gives a composite score. The cut-off score of 30 or higher indicates psychological impairment. The Y-PSC was translated in to the native language “Marathi.” Pretesting of translated scale was done on a sample of 10. Chronbach's alpha was 0.71.

### Laboratory measurements

The venous blood (random sample) was drawn from the subjects. Sample in the EDTA bulb was used for estimation of hemoglobin using Cismax XP 100 cell counter. Serum was separated from the sample in the plain bulb and used for estimation of Calcium, vitamin D, Vitamin B_1_, B_2_, B_6_, B_12_, Folic acid, Vitamin C, and zinc, levels. The serum Calcium level was estimated by modified arsenazo III method using semi auto analyser. Zinc was estimated using semi auto analyser. Vitamin D, Vitamin B_12_, and Folic acid levels were estimated using Cobas e-411 chemiluminescence. Vitamin B_1_, B_2_, and B_6_ were estimated by ELISA method. Vitamin C was estimated using Colorimetric method by Aye Kyaw (13).

### Macronutrient estimation by 24 h recall

We used 24 h recall for the dietary assessment. Nutritionist recorded each food item consumed by the subject. We used standardized cups to estimate the amount of the portion size consumed. Nutritive value of the food item consumed were estimated using the reference database (14).

All the investigations and assessments took place in the hospital on the same day.

### Statistical methods

Data has been presented as median (minimum – maximum) and as percentages. Miconutrient concentrations and estimated macronutrients, anthropomrtric measurements were tested for normality using Shapiro-Wilk test. Vitamin D, Vitamin C, height, and weight were the only normally distributed variables. All the non-normal variables were log transformed. Association between micronutrients, macronutrients, and psychosocial score was analyzed by multiple linear regression. Regression coefficient β and *p*-values were derived by linear regression using micronutrient concentration, estimated macronutrient intake as exposures and psychosocial score as a outcome. Regression coefficient β represents change in psychosocial score (outcome) per unit change in exposure variable (micronutrient or macronutrients). *p*-values were adjusted for age. We divided the micronutrient concentrations and estimated macronutrient intake of calories, protein, fat, calcium, and iron in to age specific quartiles. Those lying in the lowest quartile were tagged as having low levels of that micro or macronutrient, and the rest were tagged as having high levels. Odds ratios for psychological impairment for those with low levels were calculated together with 95% confidence intervals. Those lying completely beyond 1.0 or below 1.0 were considered as significant.

An awareness session for parents and school teachers was conducted before the start of the study and their signatures were obtained and at the time of blood collection individual parents were informed verbally. Assent of adolescent girl was also obtained.

Consent procedure and study was approved by the Ethical Committee of BKL Walawalkar Rural Medical College. Study took place in December 2016.

## Results

About 46% of girls could be classified as psychologically impaired.

### Anthropometry (Table 1A)

Median age of the girl was 14 years with a range (minimum-maximum) of 11–16 years. Based on WHO criteria 23.8% were stunted. Using IOTF classification 61% were underweight, 35% normal weight, and only 4% were obese.

**Table 1A:** Anthropometry of adolescent girls (*n* = 80).

	**Unit**	**Median (Minimum, maximum)**
Age	year	14(11, 16)
Height	cms	149.5(131, 162)
Weight	Kg	36.4(22.4, 60.6)
BMI^*^	Kg/m^2^	16.4(12.1, 27.7)

* *Log normal distribution*.

### Micronutrients (Table 1B)

About 23% girls were anemic. More than 2/3rd were calcium, zinc and folic acid deficient. About 36% were vitamin B_1_ deficient. Vitamin C deficiency was extremely low (6.6%). None of the subjects were deficient in Vitamin B_12_, Vitamin B_2_, vitamin B_6_, and Vitamin D (data not shown).

**Table 1B:** Micronutrient levels in adolescent girls (*n* = 80).

	**Unit**	**Median (Minimum, maximum)**	**Deficiency**	***n***	**%**
Haemoglobin^*^	gm%	12.1 (7.5, 14.1)	<11.5	18	22.5
Ferritin^*^	ng/mL	40 (8, 242)	<10	2	2.5
Calcium^*^	mg/dL	8.1 (7.0, 10.2)	<8.8	62	77.5
Vitamin D	mg/ml	21.0 (9.8, 30.8)	<8.2	0	0
Zinc^*^	μg/L	680 (395, 1210)	<794	54	67.5
Vitamin ${\text{B}}_{1}^{*}$	ng/mL	47.5 (0.18, 140)	<3.9	29	36.3
Vitamin ${\text{B}}_{2}^{*}$	ng/mL	27.0 (0.90, 42.0)	<0.31	80	0
Vitamin ${\text{B}}_{6}^{*}$	ng/mL	260 (2.5, 431.0)	<3.9	1	1.3
Vitamin ${\text{B}}_{12}^{*}$	pg/mL	271.3 (222.4, 305.0)	<211	0	0
Folic acid	ng/mL	0.86 (0.04, 7.86)	<2.0	67	89.3
Vitamin C	mg/dl	1.5 (0.2, 3.7)	<0.6	5	6.6

* *Log normal distribution*.

### Macronutrients (Table 1C)

Except for fat, the proportion of those with estimated calorie, protein, calcium and iron intake well below the RDA (Recommended Dietary Allowance) varied in the range of 70–90%

**Table 1C:** Macronutrient intake in adolescent girls (*n* = 80).

	**Unit**	**Median (Minimum, maximum)**	**Deficiency**	***n***	**%**
Energy^*^	Kcal	1, 086(585, 2, 180)	Below RDA	78	97.5
Protein^*^	gm	36.7(19, 104)	Below RDA	69	86.5
Fat^*^	Gm	28.9(12.0, 98.8)	Below RDA	22	27.5
Calcium^*^	Mg	189.4(49.1, 701.6)	Below RDA	79	98.8
Iron	mg	13.95(5.68, 601.42)	Below RDA	59	73.8

* *Log normal distribution*.

### Associations of micronutrient and macronutrient levels and psychosocial score (Table 2)

On multiple regression analysis calcium concentrations as well as fat and calcium intake were inversely associated with psychosocial score.

**Table 2:** Association between micronutrients, macronutrients and psychological score.

**Exposures**	**β**	**95% CI**	***P***
**MICRONUTRIENTS**			
Haemoglobin	0.33	(−1.12, 1.78)	0.65
Ferritin	−0.01	(−0.05, 0.03)	0.70
Calcium	−1.69	(−4.02, −0.64)	**0.03**
Vitamin D	−0.25	(−0.69, 0.19)	0.23
Zinc	−0.003	(−0.01, 0.01)	0.52
Vitamin B_1_	−0.001	(−0.05, 0.02)	0.48
Vitamin B_2_	0.05	(−0.13, 0.23)	0.57
Vitamin B_6_	−0.01	(−0.01, 0.01)	0.85
Vitamin B_12_	0.03	(−0.08, 0.14)	0.59
Folic acid	0.44	(−0.91, 1.69)	0.48
Vitamin C	2.01	(−0.23, 4.25)	0.08
**MACRONUTRIENTS**			
Energy	−0.09	(−0.22, 0.03)	0.15
Protein	−0.09	(−0.22, 0.03)	0.15
Fat	−0.14	(−0.26, −0.01)	**0.03**
Calcium	−0.02	(−0.04, −0.01)	**0.003**
Iron	0.01	(−0.01, 0.04)_	0.30

*β and p-values are derived by linear regression using micronutrient concentration, estimated macronutrient intake, psychological score as continuous. β represents change in psychological score per unit change in exposure variable. p-values are adjusted for age. Values in bold are statistically significant*.

### Nutrient deficiencies and psychological impairment (Table 3)

Subjects with low calcium concentrations had significantly higher psychological score when compared to those with high concentrations (29.4 vs. 25.7, *p* < 0.05). They also had significant odds ratio of psychological impairment [2.47, 95% CI (1.21, 4.31)]. Odds ratios for psychological impairment were also significant for those with low calcium and iron intake.

**Table 3:** Psychological score and odds ratios for psychological impairment for those with low and high levels of micronutrient or macronutrients.

**Exposures**	**Low**	**High**	***P* for difference**	**Odds ratios with 95% confidence interval for psychological impairment (High category is reference)**	
**MICRONUTRIENTS**				
Haemoglobin	28.7 (6.2)	28.5 (7.6)	0.92	1.43	(0.48, 4.21)
Ferritin	26.6 (8.0)	29.1 (7.0)	0.20	0.50	(0.16, 1.51)
Calcium	29.4 (6.9)	25.7 (7.7)	**0.03**	**1.47**	**(1.21, 4.31)**
Vitamin D	29.9 (10.2)	28.2 (6.2)	0.39	1.43	(0.48, 4,21)
Zinc	29.0 (9.2)	28.4 (6.7)	0.77	0.78	(0.26, 2.31)
Vitamin B_1_	28.0 (6.6)	28.8 (7.7)	0.62	0.63	(0.24, 1.64)
Vitamin B2	27.4 (8.9)	28.9 (6.8)	0.47	1.23	(0.43, 3.55)
Vitamin B6	28.7 (7.7)	28.5 (7.2)	0.95	0.78	(0.26, 2.31)
Vitamin B12	28.2 (5.9)	28.6 (7.7)	0.85	1.43	(0.48, 4.21)
Folic acid	28.9 (7.6)	28.8 (7.3)	0.94	0.91	(0.21, 3.96)
Vitamin C	28.1 (7.4)	28.7 (7.3)	0.79	1.50	(0.20, 3.70)
**MACRONUTRIENTS**				
Energy	30.8 (8.3)	27.9 (6.9)	0.24	1.94	(0.65, 5.81)
Protein	30.4 (8.3)	28.0 (6.9)	0.48	1.68	(0.55, 5.11)
Fat	29.6 (8.0)	28.2 (7.1)	0.22	1.05	(0.36, 3.11)
Calcium	30.4 (8.8)	27.7 (6.6)	**0.03**	**1.43**	**(1.08, 3.19)**
Iron	31.4(8.8)	27.7 (6.6)	**0.046**	**3.04**	**(1.02, 9.26)**

*Low, belongs to lowest age specific quartile of the exposure; High, belongs to age specific quartiles 2, 3, 4 of the exposure; Mean (SD)*.

*Values in bold are statistically significant*.

## Discussion

This is the first report from the KOKAN region of western India reporting the association between nutrition and psychological health of rural adolescent girls. A very high proportion of deficiency was observed for 3 micronutrients (zinc, calcium, and folic acid) followed by moderate deficiency of vitamin B1 and substantially low deficiency of vitamin C.

There have been many reports linking micronutrient deficiencies with psychological health of adolescents. Two studies in Iran (15, 16) found inverse association of serum zinc levels with mood disorders including depression and anxiety among adolescent females. A study in Turkey (17) found lower levels of vitamin B_12_ and vitamin D in patients with obsessive compulsive disorder compared to control group though there was no significant difference between groups in terms of folate levels suggesting the role of one carbon metabolism. A study in US (18) among Latino pregnant adolescent girls has reported the associations between micronutrients and depression. There have been some reports from India on psychological parameters among the adolescents and their link to nutritional exposures. A study in adolescents from the city of Hyderabad (19) found an association between iron nutrition, physiological deregulation, and stress. Another study (20) amongst secondary school adolescent girls in Pune city found poor cognitive performance amongst girls with zinc deficiency. In our study there was no association of zinc concentrations with psychological impairment. Similar observations have been made with respect to folic acid in a study from Delhi (21). Both of these reports come from urban settings.

We have measured total calcium concentration which is in lower range in more than 70% of the girls. Critical initial laboratory testing is needed to rule out ionic hypocalcaemia. Irritability and weakness can be correlated to mild hypocalcaemia in our subjects which we have not recorded. Also vitamin D deficiency was not observed in our study. Thus in our study with normal levels of vitamin D, the etiology of hypocalcaemia can be correlated only to nutritional deficiency. Another reason could be, due to increase growth velocity during adolescent period, the increased demand of dietary calcium is not met. Other than calcium we have measured other micronutrients which did not show association with psychological impairment despite low levels in most of them. One surprising finding in our study was lack of low vitamin B_12_ concentrations which have been reported to be very low in other regions of India (22–24). Though we have not measured folate, the folic acid (essentially a synthetic form of folate) deficiency was very high (89%). Neither vitamin B_12_ nor folic acid were associated with psychological impairment. Homocysteine plays an important role in B_12_–folate metabolism, but we have not measured homocysteine in our study. Deficiency of other B vitamins, B_2_ and B_6_ was absent in our study. Proportion of subjects with vitamin B_1_ deficiency was very high. But this needs further exploration on a larger scale. Unlike calcium there was a lack of association of other micronutrients (zinc, vitamins B_1_, and folic acid) with psychological impairment where our subjects were substantially deficient. This may suggest a role of specific micronutrient in psychological impairment.

There were many positive aspects of our study. This was the first report from the KOKAN region on the link between nutrition and psychological health of the rural adolescent girls where malnutrition is still prevalent. Yet the deficiency of 2 major micronutrients vitamins B_12_ and D was virtually absent. The negative aspects are the small sample size and lack of homocysteine measurements to explore B_12_ folate pathway. We used Y-PSC because of its simple administration. Y-PSC has been used in Nepal (25) which has similar socio cultural setting like India. We could not analyse further according to Y-PSC clusters because of small sample size. Also we were unable to explore the independent effects of each of the nutrients measured again because of small sample size. We are planning to do a study on larger scale to replicate our findings and plan to examine the effect of a particular nutrient or food on not only psychological health but also on cognitive function.

India is witnessing a rapid rise in prevalence of non-communicable diseases (NCD) like diabetes, coronary heart disease, and hypertension over last decade (26–30). In a developing country like India, where there is a lack of basic needs of life with lowest per capita income, spending money on health is beyond reach for most people. The best way to fight the epidemic of NCD's is to prevent them developing. British epidemiologist David Barker laid down the hypothesis in the 90's which says that intrauterine growth retardation, low birth weight, and premature birth have a causal relationship to the origins of hypertension, coronary heart disease, and non-insulin-dependent diabetes in the middle age (31, 32). If the risk of many NCD's in adulthood in our communities is largely determined before birth, then a critical question to be addressed is the extent to which these risks may be minimized. Poor fetal growth is most commonly diagnosed in late gestation or even after birth, and there are not yet any effective intrauterine interventions for the baby who is growing poorly. We consider that the best approach to the epidemic of NCD's in adulthood is to focus on the health of adolescent girls, as the prospective mothers of the next generation. We have demonstrated how the poor nutritional status of adolescent girls affects their psychological health. It is possible that improvement in their nutritional status using dietary, and food based interventions will lead to improvement in their own psychological health and also benefit the subsequent generations with reduced risk of NCD's in the adulthood.

## Author contributions

All authors listed have made a substantial, direct and intellectual contribution to the work, and approved it for publication.

### Conflict of interest statement

The authors declare that the research was conducted in the absence of any commercial or financial relationships that could be construed as a potential conflict of interest.

## Footnotes

^1^www.unicef.org/sowc2011/pdfs/precnapprox_EN_02092011.pdf

^2^The WHO Child Growth Standards. www.unicef.org/sowc2011/pdfs/SOWC-2011-Main-Report_EN_02092011.pdf
